# Genome-wide sequencing for the identification of rearrangements associated with Tourette syndrome and obsessive-compulsive disorder

**DOI:** 10.1186/1471-2350-13-123

**Published:** 2012-12-19

**Authors:** Sean D Hooper, Anna CV Johansson, Christian Tellgren-Roth, Eva-Lena Stattin, Niklas Dahl, Lucia Cavelier, Lars Feuk

**Affiliations:** 1 Department of Immunology, Genetics and Pathology, Rudbeck Laboratory and Science for Life Laboratory, Uppsala University, Uppsala 751 85, Sweden; 2 Department of Medical Biosciences, Medical and Clinical Genetics, Umeå University, Umeå, Sweden

**Keywords:** Tourette syndrome, Paired end sequencing, Chromosomal translocation, Structural variations

## Abstract

**Background:**

Tourette Syndrome (TS) is a neuropsychiatric disorder in children characterized by motor and verbal tics. Although several genes have been suggested in the etiology of TS, the genetic mechanisms remain poorly understood.

**Methods:**

Using cytogenetics and FISH analysis, we identified an apparently balanced t(6,22)(q16.2;p13) in a male patient with TS and obsessive-compulsive disorder (OCD). In order to map the breakpoints and to identify additional submicroscopic rearrangements, we performed whole genome mate-pair sequencing and CGH-array analysis on DNA from the proband.

**Results:**

Sequence and CGH array analysis revealed a 400 kb deletion located 1.3 Mb telomeric of the chromosome 6q breakpoint, which has not been reported in controls. The deletion affects three genes (GPR63, NDUFA4 and KLHL32) and overlaps a region previously found deleted in a girl with autistic features and speech delay. The proband’s mother, also a carrier of the translocation, was diagnosed with OCD and shares the deletion. We also describe a further potentially related rearrangement which, while unmapped in Homo sapiens, was consistent with the chimpanzee genome.

**Conclusions:**

We conclude that genome-wide sequencing at relatively low resolution can be used for the identification of submicroscopic rearrangements. We also show that large rearrangements may escape detection using standard analysis of whole genome sequencing data. Our findings further provide a candidate region for TS and OCD on chromosome 6q16.

## Background

Tourette syndrome (TS) is a spectrum of developmental neuropsychiatric disorders characterized by persistent involuntary motor and vocal tics. Tic disorders can be divided according to their duration in a transient form (<12 months) and a chronic form (>12 months). Transient tics is the mildest and most common form. The criteria for TS, according to the Diagnostic and Statistical Manual of Mental Disorders (DSM-IV), are onset before 18 years of multiple motor tics and one or more vocal tics persisting more than a year. During this period there should not have been a tic free period of more than three consecutive months. The symptoms should cause a marked distress or psychosocial impairment. The onset of TS is usually between 3–8 years of age and although the severity of tics usually declines with age, presentation of other neuropsychiatric syndromes may appear and persist with significant functional impairment [1]. The prevalence of TS varies in different studies but converges to about 1% and with a male to female ratio of 1.6-10:1 [2]. In studies of children with special needs the prevalence of TS is considerably higher and up to 30% [3]. A majority of TS patients have co-morbidities that may be more disabling than the tics [4]. Between 30% and 50% of probands with TS will experience obsessive compulsive disorder (OCD) characterized by obsessions (e.g. recurrent and disturbing thoughts) and compulsions (e.g. repetitive or ritualized behaviors). The DSM-IV criteria for OCD may include either obsessive or compulsive symptoms recognized as unreasonable by the patient. The symptoms should also cause marked distress or significantly interfere with the person’s life. The prevalence for OCD in the general population is in the range of 2-3% with a chronic course [5]. Men and women are affected equally. The high frequency of co-morbidity for OCD and TS has led to the suggestion of a shared mechanism. A shared genetic mechanism is supported by family studies showing a vertical transmission for both disorders [6]. In patients with both disorders, symptoms from OCD may precede the tics but the OC symptoms usually culminate a few years after the worst period of tics [7]. As for tics, early onset co-morbid OC symptoms decline with age and may go into remission suggesting a similar course for the two disorders [8]. For TS alone, there is now compelling evidence for the involvement of genetic factors as illustrated by family and twin studies. The concordance rate for TS in monozygotic twins is between 50% and 77%, compared to 10–23% in dizygotic twins, suggesting a genic etiology [9]. Similar figures are observed in twin studies of OCD [10]. Chromosome rearrangements, genetic linkage analysis and association studies in TS cohorts have led to suggestions of multiple candidate gene regions, specific genes and pathways (See [11] for a review). However, findings from different studies are inconsistent which has led to suggestions that there is extensive genetic heterogeneity, gene-gene interactions, sample heterogeneity as well as epigenetic models in the etiology of TS [1]. Still, rare cases of TS associated with cytogenetic rearrangements have in a few cases pointed out genes involved in the etiology of the disease and in subsets of patients. Notable examples are the combination of cytogenetic and molecular approaches that led to the identification of the SLIT and NTRK-like family member 1 *SLITRK1*[12], inner mitochondrial membrane peptidase 2 like (*IMMP2L*) [13] and contactin associated protein-like 2 *(CNTNAP2)*[14] genes. To date, of seven independent balanced reciprocal translocations reported to be associated with TS, two involve chromosome 6 [15,16]. The latter work describes a 6q21 translocation, while the location of the former is not known.

In this study, we describe a balanced cytogenetic chromosome translocation t(6;22)(q16.2;p13). The rearrangement segregates in a mother affected by OCD and her son with TS. The status of the father is unknown. In order to map the breakpoint(s) in more detail and to identify any related or additional sub-microscopic rearrangements, we performed whole genome mate-pair sequencing and high resolution SNP array analysis on the male proband. We present herein the mapping of the chromosomal breakpoints associated with TS and OCD as well as the identification of a number of additional genomic variations. Notably, the analysis revealed a 400 kb deletion on chromosome 6q in both mother and son.

## Subjects and methods

### Patients

The proband is a now 29 year old man with a history of daily motor tics, mainly head movements, with an onset at 6–7 years of age as well as daily vocal tics expressed as grunting that appeared at the age of 10 years. The symptoms have previously interfered with his daily social activities and attendance at school. The motor tics have declined with age and the vocal tics have disappeared since a few years. The proband has also a history of compulsive behavior, such as repeated check of the door and the stove before leaving the house, which is now in remission. He has cognitive functions within the normal range and he attended public school/high school with slight problems in mathematics and reading. He is now working full time. The mother, also with cognitive functions within normal range, has suffered from daily obsessive thoughts causing anxiety and distress since adolescence. The obsessions has previously interfered with her social life but has declined with age and she has been able to work full-time. She has no history of vocal or motor tics. Two sisters and the maternal grandparents of the proband refrained from participating in the study and information about other relatives were not available. Mother and son were diagnosed by psychiatrists according to the DSM-IV criteria for OCD and Tourette syndrome, respectively, without support for autistic spectrum disorder. Cytogenetic analysis revealed that both mother and son carry an apparently balanced reciprocal chromosome translocation t(6;22)(q16.2;p13). Written informed consent for participation in the study was obtained from the proband and his mother. The study was approved by the regional ethical committee of Uppsala.

### Mate-pair sequencing

Thirty μg of DNA were used to construct SOLiD3 mate-pair libraries according to the manufacturer’s instructions. Briefly, the DNA was sheared into fragments of about 3 kb by HydroShear (Genomic Solutions) and end-repaired using End Polishing Enzyme 1 and 2. Cap adaptors (5^′^-pACAGCAG-3^′^, 5^′^-CATGTCGTCp-3^′^) are ligated to both ends of the fragments. Next, the adapter ligated DNA sample was separated on a 0.8% agarose gel and DNA fragments ~3 kb in length were recovered and purified. The sizes and concentrations of adapter ligated DNA strands were quantified using a Bioanalyzer kit (DNA 7500, Agilent). The sample was circularized using a biotinylated internal adaptor, nick translated with *E.coli* DNA polymerase 1 and digested with T7 exonuclease and S1 nuclease. Digested DNA was end-repaired using End Polishing Enzyme 1 and 2 and bound on streptavidin beads. P1 (5^′^-CCACTACGCCTCCGCTTTCCTCTCTATGGGCAGTCGGTGAT-3^′^, 5^′^-ATCACCGACTGCCCATAGAGAGGAAAGCGGAGGCGTAGTGGTT-3^′^) and P2 adaptors (5^′^-AGAGAATGAGGAACCCGGGGCAGTT-3^′^, 5^′^-CTGCCCCGGGTTCCTCATTCTCT-3^′^) were ligated to the fragments. The library was further nick-translated followed by PCR-based amplification and released from the beads. PCR products were separated on a 4% agarose gel and a 250–350 bp library band was recovered, purified, and verified using a Bioanalyzer kit (Agilent, DNA 1000). Throughout the library preparation procedure, DNA was purified and concentrated with QIAquick columns (QIAGEN) after each enzymatic reaction and PCR. Emulsion PCR was performed according to the manufacturer’s manual (SOLiD3 System Templated Bead Preparation Guide, Applied Biosystems) before SOLiD sequencing. Subsequently, 50 nt mate-pair sequences were collected on the AB SOLiD3 instrument.

### Reads and filtering

In total, we obtained 85 million independent reads which were mapped to the hg18 version of the human genome, corresponding to an average coverage of 3x. A small fraction of reads mapped with the mate pairs at a distance or orientation that was deviating from the expected based on the library insert size. This group of reads was then divided into four categories; insertions, deletions, inversions and transpositions/translocations (see Additional file 1). To address the issues of false positives in all four categories of rearrangements, we designed a series of filters to remove spurious rearrangements (Table 1). First, we clustered reads showing the same chromosomal rearrangement within a distance corresponding to two insert lengths. The number of reads which support a cluster were then considered to be a measure of the quality of the prediction. We chose to focus on the rearrangements with the highest number of supporting reads because all rearrangements, and translocations in particular, are prone to false positives due to mapping errors and reference assembly artifacts. Second, we filtered out events also present in two unrelated in-house control samples without TS or OCD in order to further remove mapping artifacts or variation not relevant to the phenotype. In this step, we also removed all events from highly repetitive regions such as the telomeres or centromeres. Third, known variations based on CNVs from the Database of Genomic Variants [17] were removed. Finally, for insertions/translocations, we analyzed the positioning of anchors versus the reference genome. Essentially, we assume that a genuine translocation or transposition is characterized by a correlation in the positions of mate-paired anchors; as the upstream anchor position increases, so should the downstream anchor position. In the case of an inversion, we expect an inverse relationship between the upstream and downstream anchor. In terms of correlation between upstream position and downstream position, we expect a strong and significant positive correlation between up- and downstream anchors in case of a *p-p* or *q-q* translocation while a strong and significant negative correlation between anchors is expected in case of a *p-q* translocation. We therefore calculated the correlation coefficient between anchor positions on each chromosome in order to further exclude false positives caused by repetitive sequences from true positive inter-chromosomal rearrangements. Translocations with significant positive or negative correlation coefficients were considered more likely to be true positives. For single nucleotide variants (SNVs) we selected those supported by at least 3 reads (Table 1). These were filtered vs dbSNP [18] and the 1000 Genomes Project.

**Table 1:** Rearrangement clusters before and after filtering

**Filter**	**Insertions**	**Deletions**	**Inversions**	**Translocations**
**Unfiltered**	450	444	99	99
**vs. control**	160	148	21	12
**vs. hgvar**	76	20	2	12

Filter: Filtering levels; unfiltered rearrangements, filtered vs. controls and repetitive sequences, and filtered vs. known human genome variations (dbvar). The number of clusters with at least 13 reads for each rearrangement category is provided for each filter level. For instance, we observe 21 possible inversions after filtering vs. the control set, but 99 before filtering.

### Fluorescent in situ hybridization (FISH) analysis

Chromosome 6q specific BAC clones (Additional file 1) were purchased (BAC PAC resources, Children’s Hospital and research Center at Oakland) and used for FISH analysis on metaphase chromosomes as described [19]. Briefly, the BAC DNA of the clones was labeled by incorporation of with either Orange-dUTP or FITC-dUTP (Abbott) by nick-translation and hybridized o/n on metaphase chromosomes prepared from cultured cells from the proband. The chromosomes were counterstained with DAPI and the slides were analyzed with a Zeiss Axioscope microscope using a CCD camera and metasystems ISIS capture software.

### SNP array analysis and copy number variation

Array based detection of copy number variation was performed with the Affymetrix Genome-Wide SNP Genotyping 6.0 Array (Affymetrix Inc., Santa Clara, CA, USA). Analysis was performed using the Genotyping Console software.

## Results

Initial chromosome analysis identified a translocation between the long arm of chromosomes 6 and the short arm of chromosome 22, resulting in the karyotype t(6;22)(q16.2;p13). Validation and further mapping by fluorescence in situ hybridization analysis (FISH) narrowed down the translocation breakpoint to a 2 Mb region on chromosome 6 (at the approximate position 95–96 Mbps), flanked by BAC clones RP11-419I4 on the proximal side and RP11-482 L14 on the terminal end.

### Sequence analysis and variation detection

In order to identify the breakpoint at higher resolution, the proband DNA was sequenced using SOLiD mate-pair sequencing. A library of ~3 kb inserts was sequenced to generate approximately 85 million reads that could be aligned to the reference assembly. We started by performing a global characterization of variation in the proband to identify any candidate causative variants in addition to the known translocation. We first generated a list of single nucleotide variants (SNVs) that were supported by at least 3 sequence reads. In total, we identified 1,738,213 SNVs [20] (Table 2). Of these, 24,122 (5%) remain after filtering against dbSNP [18] and the 1000 Genomes Project. The majority (93%) of all detected SNVs are either intergenic or intronic sequences. Within coding regions we identified 116 non-synonymous SNVs all of which result in missense variants (Additional file 2: Table S1). None of the coding variants are located in genes previously associated with TS and further analysis of these may be warranted.

**Table 2:** Single nucleotide variants

**Number of SNVs**	**Total**	**Not in dbSNP**
**Total**	1,738,213	24,122
**Intergenic**	1,060,399 (61%)	14,526 (60.2%)
**Intronic**	596,487 (34%)	8,010 (33.2%)
**ncRNA**	42,414 (2%)	790 (3.2%)
**Splice site**	129 (0.01%)	0
**Down/upstream**	17,178 (1%)	346 (1.4%)
**UTR3 / UTR5**	12,979 (1%)	0
**Exonic**	8,860 (0.5%)	180 (0.7%)
**Exonic, synonymous**	4,457 (0.2%)	64 (0.2%)
**Exonic, non-synonymous**	4,367 (0.2%)	116 (0.4%)
**Exon, stop-codon**	21 (<0.01%)	0

The variants are annotated based on their positions relative to RefSeq genes. The left column includes the total number of variants and the fraction of the total number found in each annotation category. The right column indicates the corresponding numbers for variants not found in dbSNP (ver 130). UTR: untranslated region, ncRNA: non-coding RNA, Down/upstream: variant in 1Kbp region up- or downstream or transcription start site.

### Structural variation

We then used mate-paired end mapping and read depth analysis to identify structural variation. Out of 85 million independent reads, 6.3 million mapped to the reference genome at a distance or in an orientation that deviated from the expected 3Kbp. insert size. These were categorized as putative deletions, insertions (including duplications), inversions and transpositions/translocations. After a step-wise filtering process, where we selected for events represented by several fragments and removed variants present in in-house controls or databases describing normal variation (dbSNP and the Database of Genomic Variants), the number of rearrangements was reduced to 110 (summarized in Table 1 and detailed in Additional file 1).

**Figure 1:**
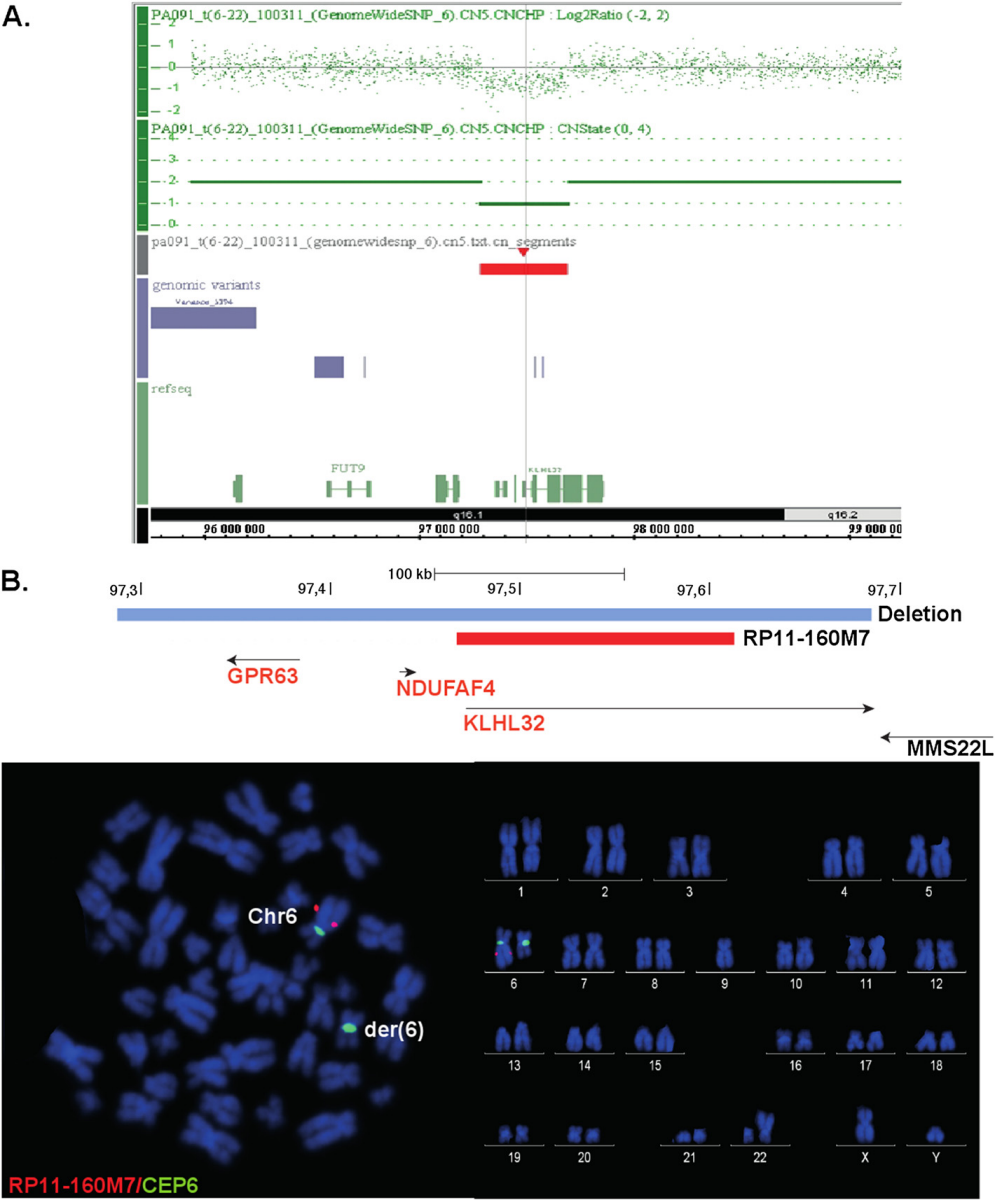
**The 400 kb deletion carried by the proband. **The deletion is mapped by SNP 6 array analysis and FISH hybridization on DNA and metaphase chromosomes, respectively. **A**. SNP 6 array data over the 6q-region showing the heterozygous deletion **B**. FISH using a BAC probe within the deletion hybridizes to the normal chromosome 6 but not to the chromosome 6 derivative.

In total, 76 insertions and 20 deletions were detected, most of which were small (<2 kb). These variants were then analyzed for overlap with annotated RefSeq (NCBI) transcripts. The largest deletion identified is located at 6q16 (at roughly 97Mbps) and spans 400 kb. Interestingly, the deletion is located on the chromosome 6 derivative and maps approximately 1.3 Mb telomeric to the translocation breakpoint identified by FISH analysis in the proband. The deletion causes a heterozygous loss of all coding sequences for the three genes *GPR63*, *NDUFA4 and KLHL32*. This result was further corroborated by CNV analysis of Affymetrix 6.0 data from the proband and the mother, as well as FISH analysis in the proband and mother (Figure 1). The sequencing analysis also revealed 20 small heterozygous deletions ranging from 2Kb to 20Kb of which two span coding sequences. These two deletions were also confirmed by homozygosity for the corresponding regions in the SNP data. One deletion resulted in a loss of one exon in the 5^′^-end of the SMAD family member 1 *(SMAD1)* gene on chromosome 4. A second deletion on chromosome 1 spans one exon of the *ATP2B4* gene encoding a Ca^2+^ATPase. Finally, an inversion spans a 9.5 kb region between the sortilin-related VPS10 domain containing receptor 1 (*SORCS1)* and X-prolyl aminopeptidase (aminopeptidase P) 1, soluble *(XPNPEP1)* genes on chromosome 10, but none of these genes have been suggested as candidates in the etiology of neuropsychiatric disease from previous studies.

Of the predicted translocations, none could be validated by FISH, suggesting that these rearrangements are more likely to be transpositions of DNA rather than real translocations. These minor rearrangements are probably artefacts of, or indicators of a normal genetic variation in individual genomes. Thus we decided to focus only on the translocation validated by FISH.

### Analysis of translocation region

No sequence read pairs were detected with homologies to both chromosomes 6 and 22 corresponding to the translocation observed by cytogenetic investigations. Since the short arm of chromosome 22 contains mainly repetitive and satellite sequences, it is primarily represented by gaps in the reference assembly, we would therefore not expect any reads bridging the translocation breakpoint to be identified. Consequently, we focused our analysis on the region surrounding the breakpoint in the q-arm of chromosome 6. As no reads were expected to map on the short arm of chromosome 22, we predicted read pairs bridging the breakpoint to have only one mapped end on chromosome 6 (orphan reads). In order to identify the translocation breakpoint, we therefore searched for a change in ratio between mapped read pairs and orphan reads. Although some regions of high fraction of orphan reads could be detected, these were also found in in-house control samples and could therefore be excluded. Our initial strategies therefore failed to identify the translocation breakpoint based on the sequencing data alone.

### Gap in the reference assembly

**Figure 2:**
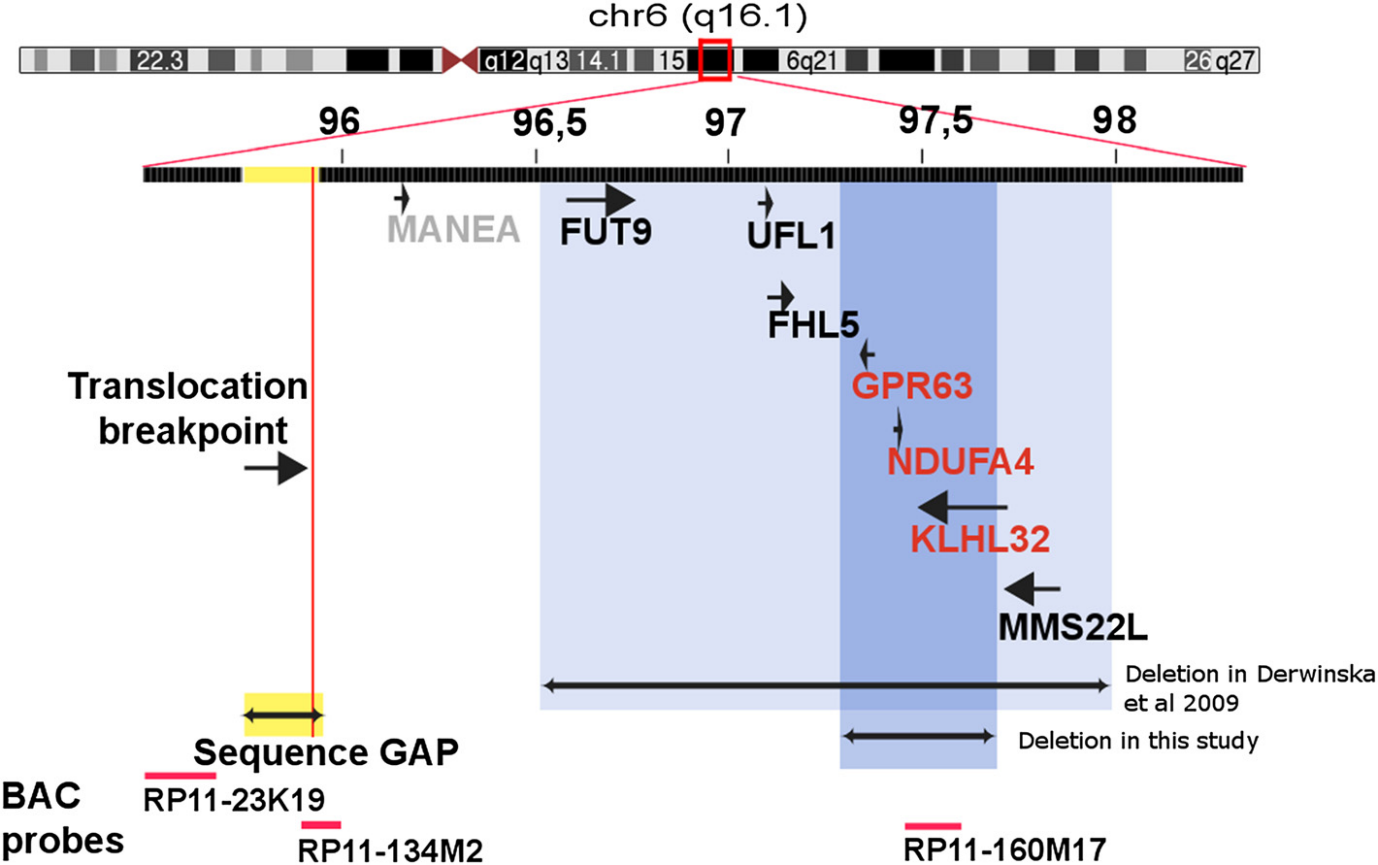
**Adapted screenshot from the UCSC genome browser **[36]**showing the rearranged 6q-region. **The FISH-probes used to characterize the rearrangements are shown in red. The figure shows a gap region of unmapped sequence, which also is the likely point for the translocation breakpoint on chromosome 6. A large (~400kbp) deletion is found telomeric of the translocation and encompasses the *GPR63*, *NDUFA4 *and *KLHL32 *genes. An overlapping deletion found in another study [32] is also displayed.

We noted that a large gap in the reference assembly (chromosome 6, near position 96Mbps) is located within the cytogenetically determined translocation breakpoint region (Figure 2), and decided to investigate this further as it could provide an explanation why no breakpoint was identified in the sequence data. We used FISH with BAC probes to further narrow down the breakpoint to a 242 kb region, which included the gap with an estimated size (in the reference assembly) of at least 200 kb. The FISH results indeed indicated that the translocation breakpoint was located inside the assembly gap. We then searched existing sources of sequence data to identify additional sequence that could be used to fill the assembly gap. Using data from the chimpanzee assembly and from the sequencing of Craig Venter’s genome [21], we identified >350 kb of sequence mapping within the assembly gap (Additional file 3: Figure S1). Through re-alignment of our sequence data, we confirmed the presence of this sequence also in the proband.

### Mapping the breakpoint

**Figure 3:**
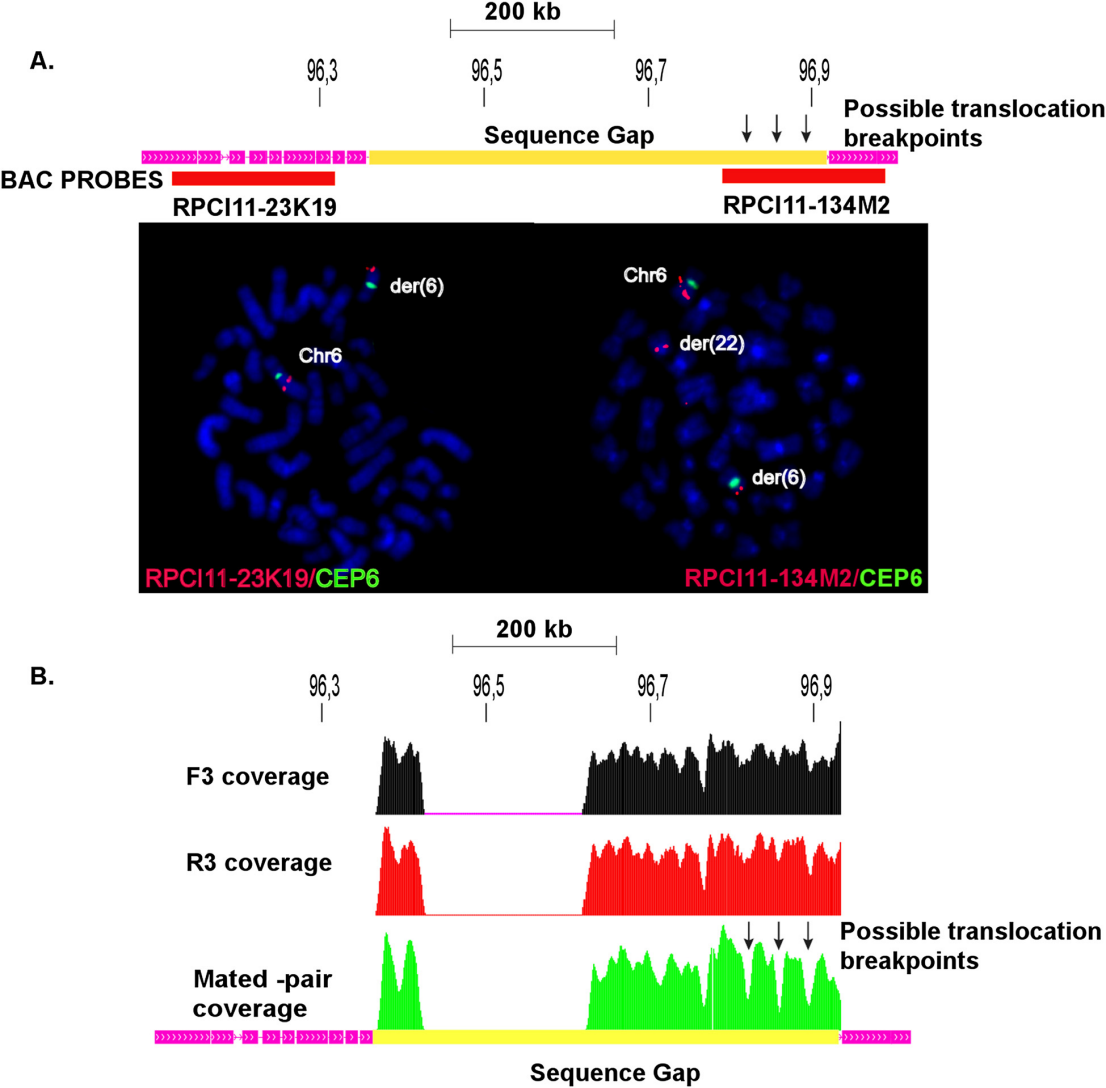
**Translocation breakpoint analysis. A**. FISH hybridization detecting the translocation breakpoint. BAC RP11-23 K19 hybridizes to chromosome 6 centromeric of the translocation breakpoint, while RP11-134 M2 hybridizes to both the derivative chromosomes 6 and 22, and encompasses the translocation breakpoint situated within the sequence gap. **B**. Sequence gap analysis using chimpanzee sequence. Reads from the TS patient were mapped to the chimpanzee genome in order to find candidate regions for the translocation within the human reference gap. In this comparison, we utilized reads where one or both ends mapped to the simian genome. If both ends mapped, we represent the number of mate-pairs spanning across each bin in the region as the green histogram. If only the forward end (i.e. the end closest to the bead) mapped, we show the frequency of mappings as the black histogram. If only the reverse end (most distant from the bead) mapped, we show the frequency as the red histogram. Thus, we can compare the number of mappings in the green histogram, which represent similar arrangements in chimp and human, to the black and green histograms which represent boundaries between shared and non-shared sequences between human and chimp. Areas with low mate-pair coverage are potential sites for the translocation breakpoint and are indicated with arrows.

In order to investigate whether the translocation breakpoint maps within the newly identified 350 kb of sequence within the assembly gap, we used BAC clones that had one end sequence anchored in the unique DNA outside the gap region, extending into the assembly gap. We first tested BAC RP11-134 M2, which has one end mapping 70 kb distal of the assembly gap. This BAC was used in metaphase FISH analysis of the proband. The results shown in Figure 3 indicate that this BAC clone spans the translocation breakpoint, showing signals on both chromosome 6 and chromosome 22 derivatives (but not on the normal chromosome 22). We could therefore determine that the breakpoint lies within the telomeric 200 kb of the >350 kb sequence gap. We then revisited our sequence data for evidence of a breakpoint by analyzing paired and unpaired reads within this region (Figure 3). This analysis identified three regions where the coverage of paired-reads decreases while that of unpaired reads remains unaffected, a pattern to be expected at a translocation breakpoint. These sequences are located 185 kb, 142 kb and 104 kb, respectively, from the end of the BAC spanning the translocation breakpoint. Unfortunately, it is difficult to determine if the unpaired reads contain chromosome 22 sequences until the short arm of chromosome 22 is fully assembled. Interestingly, the gap sequence harboring the translocation breakpoint does not contain any known genes or ESTs. Thus the 400 kb deletion, which encompasses three genes, remains our strongest candidate region for TS and OCD.

## Discussion and conclusions

We present herein the results from genome-wide mate-pair sequencing, array CGH and FISH analysis on a male patient with TS, OCD and an apparently balanced reciprocal t(6;22)(q16.2;p13). The translocation was inherited from the mother diagnosed with OCD. This observation, together with previous chromosome translocations in TS patients involving chromosome 6 and the known co-morbidity for TS and OCD, led us to hypothesize that the breakpoint on chromosome 6 and possibly additional inherited genomic variants may contribute to the neuropsychiatric phenotypes in both mother and son. To perform a genome-wide analysis of genetic variation and to map the translocation, we performed mate-pair sequencing of the proband.

Analysis of SNVs gave no strong candidates for disease association. A previous candidate gene for TS is *SLITRK1,* which has been implicated in neurite outgrowth [12,22] based on observations of rare mutations. We found one non-coding SNV in *SLITRK1* in our patient (entrezID: SNP rs41557622). The SNV is located in the 3^′^ UTR region and is also present in the 1000 Genomes Project. This variant has not been reported as associated with TS [12]. Another mutation recently linked to TS is a predicted truncation of the histidine decarboxylase *(HDC) gene*[23] encoding an L-Histidine Decarboxylase, but no exonic SNVs were found in this gene.

Analysis of CNVs in the proband’s genome was performed using the sequence data, and we detected a 400 kb deletion 1.3 Mb telomeric of the translocation 6q breakpoint region. No CNVs overlapping coding sequences in this region were described in healthy controls in either the Database of Genomic Variants or in-house control data. We confirmed by FISH that this variant is located on the translocated chr6 in both the proband and his mother. The deletion spans the G protein-coupled receptor 63 (*GPR63)*, NADH dehydrogenase (ubiquinone) 1 alpha subcomplex *(NDUFA4)* and *kelch-like 32 (KLHL32)* genes all of which are expressed in the brain*.* The *GPR63* gene is highly expressed in brain and has been suggested to act as a sphingosine 1 phosphate (S1-P) receptor [24], although this was not confirmed in following assays [25]. *NDUFA4* is a NADH:ubiquinone oxidoreductase in complex 1 of the electron transport chain in mitochondria. The gene is reported to be ubiquitously expressed, with highest expression in heart, skeletal muscle and brain [26]. *KLHL32* is highly conserved in vertebrates and expressed in the brain, but its function is poorly understood.

The proximity of the translocation and deletion on chromosome 6 suggests a potential link between the two rearrangements. It has previously been reported that apparently balanced translocations are commonly associated with additional unbalanced rearrangements in patients [27-29]. Most of these cryptic rearrangements occur at the translocation breakpoint, but there are many examples of such events occurring up to several megabases away from the translocation breakpoint [27]. The mechanism for this is not clear, but it has been suggested that replication based processes such as microhomology-mediated break-induced replication and fork stalling and template switching could give rise to complex events [30]. The formation of replications bubbles at translocation breakpoints have also been suggested to provide a basis for further rearrangements around the translocation break [31]. Determination of the mechanisms is difficult as we do not have the exact nucleotide sequence at the breakpoints, which would allow search for microhomology or larger stretches of sequence similarity. Also, we do not have access to DNA from grandparents, it is difficult to determine if the deletion occurred at the same time, or independent of, the translocation event.

Surprisingly, we were not able to identify the translocation breakpoint from the sequence data. It is noteworthy that this major genomic rearrangement, readily detected by traditional cytogenetic methods, escapes detection using genome-wide sequence analysis. We show that this can be explained by the breakpoints being located the unmapped chromosome 22p and within a gap on the human chromosome 6 reference sequence, respectively. The likelihood that a breakpoint falls in a gap is correlated with the amount of sequence present within the gap region. In this case, using different existing sequence resources, we show that at least 350 kb of sequence is missing from the human chromosome 6 reference and that the translocation breakpoint in the patient maps to within a BAC clone mapping to the gap region. The breakpoint region on chromosome 6 remains a candidate region in the etiology to TS/OCD and our observations should encourage the characterization of this gap in the reference sequence. However, the deletion stands as a stronger candidate in the etiology of TS/OCD, as it directly affects three genes.

Previous cytogenetic studies of unrelated TS patients have revealed rare cases with chromosomal rearrangements, often involving chromosome 6. Balanced reciprocal chromosome translocations may be coincidental but they may also disrupt genes or regulatory regions contributing or causing disease. This emphasizes the need to exclude or identify other gene variants in patients with such translocations. In this study, we have used a combination of whole genome sequencing, array analysis, FISH and cytogenetics illustrating methodological advantages and pitfalls in search of genomic variants associated with TS and OCD. The combination of methods used in our study suggests that current sequencing technologies cannot fully replace conventional methods in search for certain genomic variants, specifically those occurring in regions without reference sequences.

Intriguingly, the deletion found in our patient also coincides with a rearrangement previously reported in a girl diagnosed with Autism Spectrum Disorder (ASD) and developmental delay [32]. In that report, the patient carries a translocation with an associated deletion including all three genes located within the region identified in our patient (*GPR63, NDUFA4* and *KLHL32*) along with *FUT6* and *FHL5*. A link between TS and ASD is supported by a reported co-morbidity of 6.5-11% for TS in cohorts of ASD patients [33,34]. A shared genetic mechanism for the two disorders is previously illustrated by a deletion of the neuroligin 4 *(NLGN4)* gene as well as disruptions of the *CNTNAP2* gene observed in both ASD and TS [35]. In our study the proband and his mother did not present with ASD but we cannot exclude that subgroups of patients with TS or ASD may carry identical gene variants that manifest differently. In conclusion, we consider the 400 kb deletion a strong candidate involved in the etiology of TS and OCD and we provide further support for a connection between TS and ASD centered on chromosome 6.

## Competing interests

The authors declare that they have no competing interests

## Authors' contributions

LC, ND and LF designed the study, LC and LF performed the experiments, SDH, CTR and ACVJ analyzed the data, ELS provided clinical data and analysis. LC, LF, SDH and ND wrote the manuscript. All authors read and approved the final manuscript.

## Pre-publication history

The pre-publication history for this paper can be accessed here:

http://www.biomedcentral.com/1471-2350/13/123/prepub

## Supplementary Material

Additional file 1 **This section explains the tables in tabs insertions, deletions, inversions and translocations. **We have also added a figure describing the basics of paired end sequencing for reference. Click here for file

Additional file 2 **Table S1. **Missense singe nucleotide mutations not present in dbSNP or 1000 genomes. Click here for file

Additional file 3 **Figure S1. **Identification of HuRef (Venter) and Celera contigs across the human gap region. The sequences corresponding to the human gap are evident from the “Human chain” track at the bottom of the figure. Each red bar in the top part of the figure corresponds to an accessioned sequence from Celera or HuRef. Accessions AADB02010123 and ABBA01014539 seem to span the gap in chimpanzee (as shown in the BLAT result below in the figure). Approximately 1000 bp of sequence in this contig does not align to chimp (or human). This may indicate that the gap is very small in chimpanzee. However, this is contradicted by the fact that no mate-pairs span across the gap. It is possible that the highly repetitive sequences adjacent to the chimpanzee gap leads to poor or erroneous sequence alignment. Click here for file
