# Supplementary material for: Genome-wide sequencing for the identification of rearrangements associated with Tourette syndrome and obsessive-compulsive disorder
Source: BMC Med Genet. 2012 Dec 19;13:123. doi: 10.1186/1471-2350-13-123 (PMC3556158; doi:10.1186/1471-2350-13-123)

**Supplementary Figure 1**

Identification of HuRef (Venter) and Celera contigs across the human gap region. The sequences corresponding to the human gap are evident from the “Human chain” track at the bottom of the figure. Each red bar in the top part of the figure corresponds to an accessioned sequence from Celera or HuRef. Accessions AADB02010123 and ABBA01014539 seem to span the gap in chimpanzee (as shown in the BLAT result below in the figure). Approximately 1000bp of sequence in this contig does not align to chimp (or human). This may indicate that the gap is very small in chimpanzee. However, this is contradicted by the fact that no mate-pairs span across the gap. It is possible that the highly repetitive sequences adjacent to the chimpanzee gap leads to poor or erroneous sequence alignment.


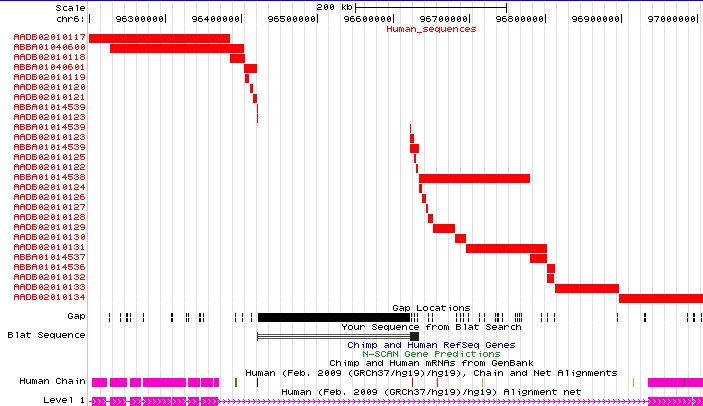

Supplement: Additional file 3 — Figure S1. Identification of HuRef (Venter) and Celera contigs across the human gap region. The sequences corresponding to the human gap are evident from the “Human chain” track at the bottom of the figure. Each red bar in the top part of the figure corresponds to an accessioned sequence from Celera or HuRef. Accessions AADB02010123 and ABBA01014539 seem to span the gap in chimpanzee (as shown in the BLAT result below in the figure). Approximately 1000 bp of sequence in this contig does not align to chimp (or human). This may indicate that the gap is very small in chimpanzee. However, this is contradicted by the fact that no mate-pairs span across the gap. It is possible that the highly repetitive sequences adjacent to the chimpanzee gap leads to poor or erroneous sequence alignment. [file 1471-2350-13-123-S3.doc]
